# Impact of HIV exposure without infection on hospital course and mortality among young children in sub-Saharan Africa: a multi-site cohort study

**DOI:** 10.1186/s12916-024-03790-5

**Published:** 2024-12-03

**Authors:** Christopher Lwanga, Peace Aber, Kirkby D. Tickell, Moses M. Ngari, John Mukisa, Michael Atuhairwe, Lindsay Brown, Ezekiel Mupere, Isabel Potani, Lubaba Shahrin, Brooks Morgan, Benson O. Singa, Victoria Nankabirwa, Richard K. Mugambe, Zakaria Mukasa, Judd L. Walson, James A. Berkley, Christina L. Lancioni

**Affiliations:** 1 Uganda-Case Western Reserve University Research Collaboration, Kampala, Uganda; 2 The Childhood Acute Illness and Nutrition (CHAIN) Network, Nairobi, Kenya; 3 Department of Global Health, University of Washington, Seattle, USA; 4 KEMRI/Wellcome Trust Research Programme, Kilifi, Kenya; 5 School of Public Health, Oregon Health and Science University, Portland, OR USA; 6 Department of Paediatrics and Child Health, Makerere University College of Health Sciences, Kampala, Uganda; 7 University of Toronto, Toronto, Canada; 8 International Centre for Diarrheal Disease Research, Dhaka, Bangladesh; 9 Kenya Medical Research Institute, Nairobi, Kenya; 10 School of Public Health, Makerere University College of Health Sciences, Kampala, Uganda; 11 Makerere University-Johns Hopkins University Research Collaboration, Kampala, Uganda; 12 Departments of International Health, Medicine and Pediatrics, Bloomberg School of Public Health, Johns Hopkins University, Baltimore, USA; 13 Centre for Tropical Medicine & Global Health, Nuffield Department of Medicine, University of Oxford, Oxford, UK; 14 Department of Pediatrics, Oregon Health and Science University, CDRC-P, 707 SW Gaines St, Portland, OR 97239 USA

**Keywords:** HIV exposure, Mortality, Nutritional status, Hospital length of stay

## Abstract

**Background:**

Although mortality risk associated with HIV is well described, HIV-exposed uninfected (HEU) young children are also at increased risk of hospitalization and death as compared to HIV-unexposed uninfected (HUU) children. The drivers of poor outcomes among HEU children remain unknown, limiting the development of interventions to support this vulnerable population.

**Methods:**

We performed a secondary analysis of data from a large multi-country prospective cohort [Childhood Acute Illness and Nutrition (CHAIN) Network] study. Data from 5 sites in Uganda, Kenya, and Malawi were included. Hospitalized children aged 2–23 months were followed from an index admission for 6 months after discharge to determine acute and long-term outcomes. Using perinatal HIV exposure (HEU and HUU) as the primary exposure and adjusting for child, caregiver, and household characteristics, we compared inpatient and 30-day survival outcomes, nutritional status, hospital length of stay, illness severity, and utilization of inpatient resources.

**Results:**

We included 1486 children: 217 HEU and 1269 HUU. HEU children had an increased risk of mortality both during hospitalization [adjusted OR 1.96, 95% CI (1.14–3.37)] and in the 30 days following hospital admission [adjusted hazard ratio 2.20, 95% CI (1.10–4.42)]. Wasting and stunting were more frequent in HEU than HUU children, with adjusted OR 1.41, 95% CI (1.03–1.95) and adjusted OR 1.91, 95% CI (1.34–2.70), respectively. HEU children were also more likely to have a prolonged hospital stay compared to HUU children [adjusted OR 1.58, 95% CI (1.08–2.29)], although admission diagnoses, illness severity at admission, and use of inpatient resources (supplemental oxygen, nasogastric tube, and second-line antibiotics) did not differ significantly between groups.

**Conclusions:**

HEU children are more likely to die during hospitalization and within 30 days of admission, to be wasted and stunted upon hospital admission, and to require a prolonged hospital stay, as compared to HUU children. Hospitals in settings with a high prevalence of women-living-with-HIV should ensure that maternal HIV status is established among children requiring admission and build capacity to provide additional hospital monitoring and early post-discharge support for HEU children.

**Supplementary Information:**

The online version contains supplementary material available at 10.1186/s12916-024-03790-5.

## Background

Successful prevention of mother-to-child transmission (PMTCT) of HIV infection has led to a dramatic decrease in the number of children with perinatally acquired HIV infection worldwide [1]. Given the persistently high prevalence of women of childbearing age who are living-with-HIV in some populations, there has been a subsequent increase in children who are HIV-exposed but uninfected (HEU) [2]. In 2022, there were an estimated 15.4 million HEU children, with close to 90% residing in sub-Saharan Africa [3]. Five countries account for over 50% of HEU children, including South Africa (23.8%), Uganda (7.5%), Mozambique (6.6%), Tanzania (6.1%), and Nigeria (6.0%) [4].

HEU children, particularly in the first 2 years of life, are more vulnerable to poor health outcomes [5–7] as compared to HIV-unexposed uninfected (HUU) children. Studies performed both prior to and following widespread access to antiretroviral therapy (ART) demonstrate that HEU children are at higher risk of mortality compared to HUU children [8–11]. The vulnerability of HEU children is not restricted to low-and-middle-income countries (LMIC) but is also seen in better-resourced settings such as the USA [12]. However, drivers of morbidity and mortality among HEU children remain unclear, a knowledge gap that hampers efforts to improve outcomes. Currently, there are limited international guidelines to support the provision of additional resources to this vulnerable and growing population of children.

Multiple unique exposures and immunologic abnormalities have been described among HEU children during the first years of life that may predispose them to morbidities and death [13–15]. These include intrauterine and postnatal exposure to ART, intrauterine and postnatal exposure to chronic viral infections (in addition to HIV) such as CMV, maternal dysbiosis and metabolic derangements impacting fetal and post-natal immune development, reduced duration of breastfeeding, and compromised transfer of protective maternal antibodies during gestation and breastfeeding. Additionally, HEU children are at increased risk of *Mycobacterium tuberculosis* infection which may contribute to poor growth and early mortality [5]. There is also evidence that universal risk factors for early childhood morbidity, including poor birth outcomes (preterm birth and low birth weight), suboptimal breastfeeding, and maternal mortality and poverty, occur more frequently in HEU children compared to HUU children [13–16]. The social and economic impact of living with HIV on families may also influence childhood health outcomes. For example, parents-living-with-HIV may experience more episodes of illness and visits to medical providers that have significant economic impacts. An income-earning partner may not be able to work or may experience premature death. Mothers-living-with-HIV may be at risk for increased psychological stress, depression, and stigmatization [17] that negatively impact their capacity to care for their children and place households at increased risk for malnutrition and illness [18].

There have been limited studies that examine risk factors for poor outcomes during hospitalization among HEU children, as compared to those who are HUU. For example, nutritional status has long been recognized as key to healthy childhood growth, neurodevelopment, and survival during severe illness [19, 20]. Undernutrition puts children at greater risk of dying from common infections, increases the frequency and severity of such infections, and delays recovery [21]. The impact of HIV exposure on early childhood nutritional status and mortality risk remains unclear [8, 10] and could be mediated by both biologic and social influences. Illness severity is directly related to mortality and prolonged hospitalization that may put surviving children at risk of nosocomial infections, colonization with resistant pathogens, and is also associated with increased costs to the family. We explored the associations between HIV exposure without HIV infection and inpatient and 30-day mortality, nutritional status, illness severity, utilization of hospital resources (supplemental oxygen, nasogastric tube, and second-line antibiotics), and hospital length of stay, among young children hospitalized in Kenya, Uganda, and Malawi.

## Methods

### Study design and participants

Here we present a secondary analysis of data collected during the Childhood Acute Illness and Nutrition (CHAIN) cohort study. The CHAIN study enrolled children 2 to 23 months old at the time of admission to hospital for any acute illness (excluding trauma, poisoning, and complications of congenital defects), at nine sites across sub-Saharan Africa and South East Asia, between November 2016 and January 2019 [22]. In this analysis, we examined data from five CHAIN sites with a high prevalence of women-living-with-HIV: Kampala (Uganda), Blantyre (Malawi), Migori (Kenya), Mbagathi (Nairobi, Kenya), and Kilifi (Kenya). To ensure representation of all nutritional status, and to increase inclusion of children at high risk for poor outcomes, children were recruited in three strata of no wasting, moderate wasting, and severe wasting/kwashiorkor (nutritional edema) in ratio 2:1:2. Details of the CHAIN cohort study have been previously published [22]. In brief, children were followed daily from hospital admission through discharge, and then at scheduled follow-up assessments 45, 90, and 180 days following hospital discharge. For this secondary analysis, data collected from hospital admission through discharge and up to 30 days from the date of admission were included. Both inpatient mortality and deaths within 30 days from hospital admission (to capture deaths that occurred during the early post-discharge period) were examined.

### HIV testing and classifications

All children and their mothers underwent rapid diagnostic testing for HIV infection at study enrollment using either the Alere 2, Determine HIV 1/2, or Uni-gold HIV 1/2, rapid tests. Manufacturer recommended standard operating procedures were followed. The rapid test was repeated to confirm a positive result, and/or if the results were invalid. Children with positive rapid tests, and all children < 18 months old born to mothers-living-with-HIV, had HIV DNA-PCR confirmatory testing performed. A child was considered HUU if both child and maternal HIV rapid test results were negative. A child was considered HEU if their mother was known to be living-with-HIV while pregnant and the child’s confirmatory HIV test was negative, and/or a child < 18 months old had positive HIV rapid tests and the child’s confirmatory HIV DNA-PCR test was negative. Children with positive HIV rapid tests and positive HIV DNA-PCR were considered to be living-with-HIV. If a child’s mother was not available for HIV testing, HIV exposure could be confirmed by verbal report from a caregiver that the child’s mother was known to be living-with-HIV during pregnancy. Only HEU and HUU children were included in this secondary analysis. Children confirmed to be living-with-HIV, and children with unknown HIV infection or exposure status, were excluded.

### Anthropometric assessments

Anthropometry (weight, length, mid-upper-arm-circumference/MUAC) was assessed at admission and discharge. Measurements were performed by two trained study staff following standardized protocols and using calibrated scales, length boards, and MUAC tapes provided by UNICEF. Nutritional status was determined by MUAC, using the following definitions: not wasted, MUAC ≥ 12.5 cm (age ≥ 6 months) or MUAC ≥ 12 cm (age < 6 months); moderate wasting, MUAC 11.5 to < 12.5 cm (age ≥ 6 months) or MUAC 11 to < 12 cm (age < 6 months); and severe wasting or kwashiorkor, MUAC < 11.5 cm (age ≥ 6 months) or MUAC < 11 cm (age < 6 months) or bilateral pedal edema not explained by other medical causes. For this specific analysis, nutritional status is reported as wasting and stunting. For wasting, two nutritional categories were created: (1) not wasted, MUAC ≥ 12.5 cm (age ≥ 6 months) or MUAC ≥ 12 cm (age < 6 months); (2) wasted, MUAC < 12.5 cm (age ≥ 6 months) or MUAC < 12 cm (age < 6 months) or bilateral pedal edema not explained by other medical causes. Stunting was defined as a height for age *Z* score < − 2 standard deviations from the WHO child growth standards median.

### Data collection, clinical, and socioeconomic variables

Detailed clinical data were prospectively collected at hospital admission; social and demographic data were collected within the next 48 h using standardized tools. Admission diagnosis(es) was recorded, as was receipt of medications such as antibiotics and/or traditional medications prior to presentation. As previously reported, an illness severity score was assigned to each child at hospital admission based on the presence of features of systemic inflammatory response syndrome (SIRS) [23]. The four criteria included heart rate low (< 90) or high (> 180)/min; axillary temperature low (< 36 °C) or high (≥ 38.5 °C); respiratory rate high (> 34 breaths per minute); and WBC low (< 5 × 10^9^/L) or high (> 17.5 × 10^9^/L) [24]. Illness severity scores ranged from 0 to 4 (0 = none, 1 = one sign, 2 = two signs, 3 = three signs, 4 = all four signs). Subsequently, illness severity was collapsed into a binary variable [24]: none or one sign (low illness severity) versus two or more signs (high illness severity).

All enrolled children were assessed daily throughout their hospitalization to assess signs of clinical progress or deterioration, such as the presence of WHO danger signs [25], and to monitor the use of inpatient resources including supplemental oxygen, feeding tubes, and second-line antibiotics. Length of hospital stay was computed by obtaining the number of days from admission to discharge or death. Dates, times, and presumed causes of inpatient deaths were collected from medical files and healthcare providers. For children who left hospital against medical advice prior to discharge, length of stay was computed by obtaining the number of days from admission to the date they left hospital. In addition to deaths occurring while inpatient, deaths that occurred post-discharge but within 30 days of admission to hospital were also captured (30-day mortality) through verbal contact with parent or guardian, and/or review of hospital records for children who died during a re-admission [11].

Demographic and socioeconomic data included the vital status of the child’s biological mother, caregiver age, weight and height, and education level. Standardized questionnaires were used to assess household food security and wealth index. A set of eight questions from the Food Insecurity Experience Scale (FIES) was adapted and asked to caregivers to assess household food insecurity [11]. A categorical variable defining food insecurity was created with a score of 0–3, 4–6, and 7–8 defined as low, moderate, and severe food insecurity, respectively. Appropriate diet variable was defined as exclusively breastfed for children < 6 months, more than or equal to two food groups and breastmilk for children 6 to 9 months, and more than or equal to four food groups plus breastmilk for children 10 to 23 months. Assessment of household ownership of assets such as a television or bicycle, and housing structure were adapted from the Demographic and Health Survey (DHS). Assets and housing structure variables were then used to derive the household asset index using principal component analysis (PCA). Variables with a percentage of missing data of less than 10% were imputed using the iterative PCA method before running PCA on complete observations. Asset quintiles were expressed in terms of quintiles with five categories depicting from the poorest to the least poor with each category representing approximately 20% of the participants.

All data were recorded in paper case report forms by trained staff, reviewed by site study coordinators prior to entry into a Research Electronic Data Capture (REDCap) database, and underwent validation checks by the CHAIN Network data management team.

### Statistical analysis

All available data from hospitalized HEU and HUU children at the Ugandan, Kenyan, and Malawi CHAIN sites were included, and children documented to be living-with-HIV were excluded. A priori power calculations were not performed. The main exposure variable considered was perinatal HIV exposure, with analysis focusing on HEU and HUU children. The study outcomes were inpatient mortality, 30-day mortality, nutritional status at admission, illness severity at admission, hospital length of stay, presence of danger signs, and utilization of inpatient resources (supplemental oxygen, feeding tubes, and second-line antibiotics). All the analyses were weighted to reflect the stratification by nutritional status using inverse weights (1, 0.40, and 0.39 for the not wasted, moderately wasted, and severely wasted and/or kwashiorkor, respectively) as explained elsewhere [11]. Children’s characteristics at the time of admission to hospital stratified by HIV exposure category (HEU and HUU) are reported.

For all outcomes, directed acyclic graphs (DAGs) were developed to portray relationships between biological, social, and demographic variables hypothesized to interact with each outcome. Each DAG delineates confounders, mediators, and effect modifiers, and their relationships between HIV exposure and outcome (Additional file 1: Fig. S1, S2, S4–7) [26]. Variables selected for consideration included age group (< 6 months, 6–11 months, and 12–24 months), biological sex, breastfeeding status, prematurity, nutritional wasting, limited household assets, high food insecurity, mother as primary caregiver, prolonged travel time to hospital, and recruitment site. Age < 6 months was tested as a potential effect modifier for each outcome (using a multiplicative interaction term between age and HIV exposure), and stratification was performed if a significant interaction (*p* ≤ 0.05) was identified. Subsequent analytic models were developed for each outcome to support adjustment for confounding variables, as detailed in the tables and Additional file 1.

For inpatient mortality, days-to-death between HIV exposure groups were compared using Wilcoxon rank sum test. To assess the effect of HIV exposure on inpatient mortality, a logistic regression model was used. For 30-day mortality, a multilevel parametric survival regression model with a Weibull probability distribution (as previously reported) [11] was performed. Adjustments were performed as described for inpatient mortality, with the exception that site adjustment was performed using a random-effects approach to be consistent with our prior work. An exploratory analysis that added adjustment for nutritional wasting as a confounding variable for both inpatient and 30-day mortality was also performed.

To assess the relationship between HIV exposure and nutritional status (wasted vs not wasted and stunted vs not stunted) at hospital admission, a logistic regression model was used.

To assess the relationship between HIV exposure and illness severity (low versus high), stratification based on age group (< 6 months versus ≥ 6 months) was performed as age < 6 months was found to have a significant interaction with HIV exposure (*p* = 0.013). Age-stratified logistic regression models were then applied.

To assess the relationship between HIV exposure and prolonged hospitalization, the duration of hospitalization was categorized into a binary variable using the median length of hospital stay (5 days) as the cut-off: short and prolonged duration of hospital stay. Length of hospital stay analysis was restricted to survivors and also excluded those who left against medical advice or absconded. A logistic regression model was then applied.

To assess the effect of HIV exposure on the occurrence of daily danger signs and resource utilization (oxygen use, nasogastric tube use, and antibiotic switch), days with any danger sign or the use of a specific resource were counted. A zero-inflated negative binomial regression model was applied because the days with a danger sign or use of any resource had leading zeros and was over dispersed. Zero-inflated negative binomial regression was conducted for danger signs and each resource separately.

All statistical analyses were conducted using STATA College Station TX version 15.0 and the level of significance was assessed using two-tailed *α* < 0.05 or 95% CIs.

### Ethical considerations

Ethical approval was obtained from the Oxford Tropical Research Ethics Committee and each site-specific institutional review board: Scientific & Ethical Review Unit (SERU), Kenya Medical Research Institute in Kenya; Makerere University School of Biomedical Sciences Research Ethics Committee in Uganda; and COMREC, Kamuzu University of Health Sciences in Malawi. Written, informed consent was obtained from caregivers of all study participants in their preferred local language.

## Results

### Description of study participants

**Fig. 1:**
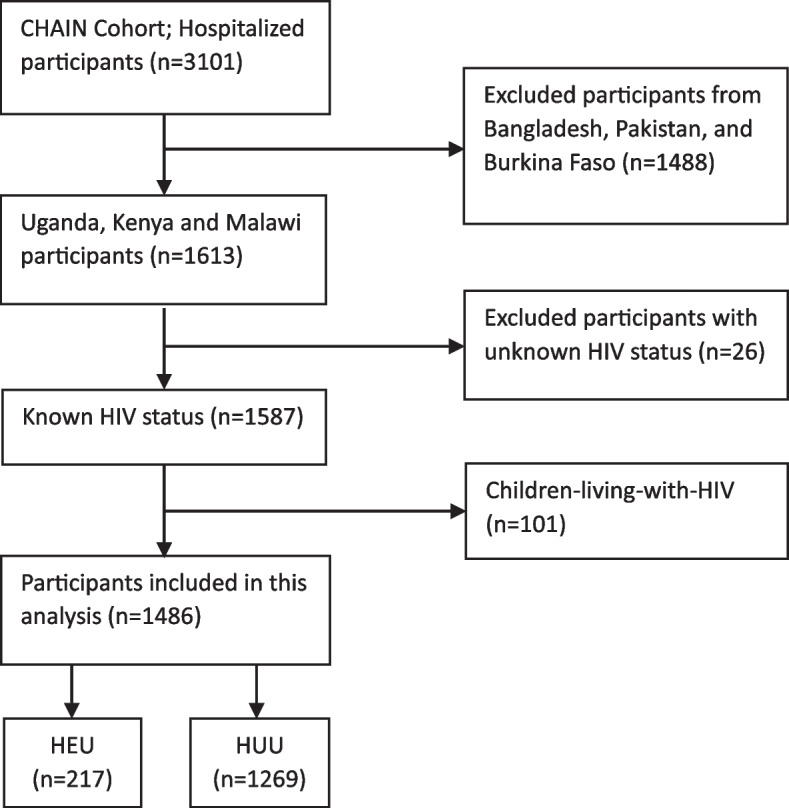
Flow diagram demonstrating children included in the analysis

Out of 3101 hospitalized children in the CHAIN cohort study from all sites, 1488 participants from sites with very low baseline HIV prevalence (Bangladesh, Karachi, and Burkina Faso) were excluded from this analysis (Fig. 1). Further, 26 participants with unknown HIV status and 101 children-living-with-HIV were excluded. A total of 1468 children were included in this analysis: 1269 HUU and 217 HEU children.

### Baseline characteristics of hospitalized children

Tables 1 and 2 illustrate the baseline characteristics of participating children and their caregivers. Overall, 55.3% of the participants were male and the median age was 11.2 months. Only 7% of children were reported as born prematurely, 7% did not have their biological mother as the primary caregiver, and 3% of biological mothers were reported as deceased. Sixty-seven percent of all children were breastfeeding at hospital admission. Among children not breastfeeding at admission, HEU children were more likely to have stopped breastfeeding by 12 months of age (Additional file 1: Table S1). Most mothers (95%) were 18–50 years of age, and over half had attained primary level as their highest level of education. The Kampala site contributed the most children to the study population.

**Table 1:** Child demographic and clinical characteristics at the time of hospital admission categorized by HIV exposure group

Characteristic	HUU	HEU	Total	*p* value
Sex				
Male	705 (55.6)	117 (53.9)	822 (55.3)	0.654
Female	564 (44.4)	100 (46.1)	664 (44.7)	
Age group				
12 and above	581 (45.8)	97 (44.7)	678 (45.6)	0.875
6 to 11	472 (37.2)	80 (36.9)	552 (37.2)	
< 6 months	216 (17.0)	40 (18.4)	256 (17.2)	
Site				
Kilifi	217 (17.1)	12 (5.53)	229 (15.4)	** < 0.001**
Mbagathi	244 (19.2)	27 (12.4)	271 (18.2)	
Migori	198 (15.6)	56 (25.8)	254 (17.1)	
Kampala	387 (30.5)	59 (27.2)	446 (30.0)	
Blantyre	223 (17.6)	63 (29.0)	286 (19.3)	
Birth weight				
Median (IQR)	3.1 (0.90)	3.2 (0.90)	3.1 (0.90)	0.698
Mean (SD)	3.1 (0.75)	3.1 (0.68)	3.1 (0.74)	
Born premature				
No	1178 (92.8)	208 (95.9)	1386 (93.3)	0.1
Yes	91 (7.2)	9 (4.2)	100 (6.7)	
Breastfeeding (any)				
No	373 (29.4)	113 (52.1)	486 (32.7)	** < 0.001**
Yes	896 (70.6)	104 (47.9)	1000 (67.3)	
Appropriate diet (all)				
No	668 (52.6)	153 (70.5)	821 (55.2)	** < 0.001**
Yes	601 (47.4)	64 (29.5)	665 (44.8)	
Age				
< 6 months				
No	121 (56.0)	28 (70)	149 (58.2)	0.1
Yes	95 (44.0)	12 (30)	107 (41.8)	
6–9 months				
No	107 (34.7)	27 (46.6)	134 (36.6)	0.087
Yes	201 (65.3)	31 (53.4)	232 (63.4)	
10–23 months				
No	440 (59.1)	98 (82.4)	538 (62.3)	** < 0.001**
Yes	305 (40.9)	21 (17.6)	326 (37.7)	
Wasting				
No	522 (41.1)	77 (35.5)	599 (40.3)	0.117
Yes	747 (58.9)	140 (64.5)	887 (59.7)	
Stunting				
No	668 (52.8)	83 (38.2)	751 (50.7)	** < 0.001**
Yes	596 (47.2)	134 (61.8)	730 (49.3)	
Used traditional medicine in last 7 days				
Yes	75 (5.9)	15 (6.9)	90 (6.1)	0.567
Used antibiotics in last 7 days				
Yes	516 (40.7)	88 (40.5)	604 (40.6)	0.976
Diagnosis of pneumonia at admission				
Yes	500 (39.4)	78 (35.9)	578 (38.9)	0.334
Diagnosis of gastroenteritis at admission				
Yes	436 (34.4)	82 (37.8)	518 (34.9)	0.327
Diagnosis of sepsis at admission				
Yes	264 (20.8)	47 (21.7)	311 (20.9)	0.775
Diagnosis of malaria at admission				
Yes	188 (14.8)	32 (14.7)	220 (14.8)	0.979
High illness severity at admission				
Yes	474 (37.4)	83 (38.3)	557 (37.5)	0.801

Data presented as *n* (%), mean (SD), and median (IQR), *n*—frequency. Appropriate diet variable was defined as follows: exclusively breastfed for children <6 months, more than or equal to two food groups and breastmilk for children 6 to 9 months, and more than or equal to four food groups plus breastmilk for children 10 to 23 months [11]. High illness severity is defined as the presence of two or more features of systemic inflammatory response syndrome (SIRS) [22]. The four features included heart rate low (<90) or high (>180)/min; axillary temperature low (<36 °C) or high (≥38.5 °C); respiratory rate high (>34 breaths per minute); and WBC low (<5 × 10^9^/L) or high (>17.5 × 10^9^/L)

**Table 2:** Caregiver and household characteristics at the time of hospital admission, categorized by HIV exposure group

Characteristic	HUU	HEU	Total	*p* value
Caregiver age				
< 18	41 (3.2)	9 (4.2)	50 (3.4)	0.638
18 to 50	1209 (95.3)	206 (94.9)	1415 (95.2)	
> 50	19 (1.5)	2 (0.9)	21 (1.4)	
Caregiver BMI				
Underweight	83 (6.5)	23 (10.6)	106 (7.1)	**0.02**
Normal	796 (62.7)	144 (66.4)	940 (63.3)	
Overweight and obese	390 (30.7)	50 (23.0)	440 (29.6)	
Caregiver education level				
Secondary/above	544 (42.9)	88 (40.6)	632 (42.5)	0.811
Primary	644 (50.8)	115 (53.0)	759 (51.1)	
None	81 (6.4)	14 (6.4)	95 (6.4)	
Biological mother alive				
No	41 (3.2)	7 (3.2)	48 (3.2)	0.997
Yes	1228 (96.8)	210 (96.8)	1438 (96.8)	
Biological mother is the primary care giver				
No	92 (7.3)	13 (6)	105 (7.1)	0.504
Yes	1177 (92.7)	204 (94)	1381 (92.9)	
Hospital travel time				
< 1 h	542 (44.0)	105 (49.1)	647 (44.7)	0.284
1–2 h	523 (42.4)	82 (38.3)	605 (41.8)	
> 2 h	168 (13.6)	27 (12.6)	195 (13.5)	
Food insecurity				
Low	598 (47.1)	71 (32.7)	669 (45.0)	** < 0.001**
Moderate	455 (35.9)	89 (41.0)	544 (36.6)	
High	216 (17.0)	57 (26.3)	273 (18.4)	
Household assets				
Poorest	311 (24.5)	55 (25.4)	366 (24.6)	**0.026**
Second	221 (17.4)	54 (24.9)	275 (18.5)	
Middle	317 (25.0)	53 (24.4)	370 (24.9)	
Fourth	278 (21.9)	42 (19.4)	320 (21.5)	
Least poor	142 (11.2)	13 (6.0)	155 (10.4)	

Data presented as *n* (%), *n*—frequency

*Abbreviations*: *HUU* HIV Unexposed uninfected, *HEU* HIV Exposed uninfected, *BMI* Body mass index

Sex, child’s age group, birth weight, and caregiver’s education level were comparable between HIV exposure categories. In the unadjusted analysis presented in Table 1, HEU children were more likely to be stunted, less likely to be breastfeeding, and less likely to be receiving an age-appropriate diet as compared to HUU children. Forty-seven percent of HUU children had a low food insecurity score, with 17% reporting high food insecurity. In comparison, the highest proportion of HEU children (41%) had a moderate score of food insecurity, with 27% reporting high food insecurity. Household assets were also significantly different between families with HEU versus HUU children, with HEU children less likely to come from the least poor households. Caregiver BMI was also significantly different, with the caregivers of HEU children more likely to be underweight and less likely to be overweight or obese, as compared to the caregivers of HUU children. There were no statistically significant differences in the admission diagnoses, use of antibiotics or traditional medications prior to admission, or illness severity at admission between HEU and HUU children.

### HIV exposure and mortality

**Fig. 2:**
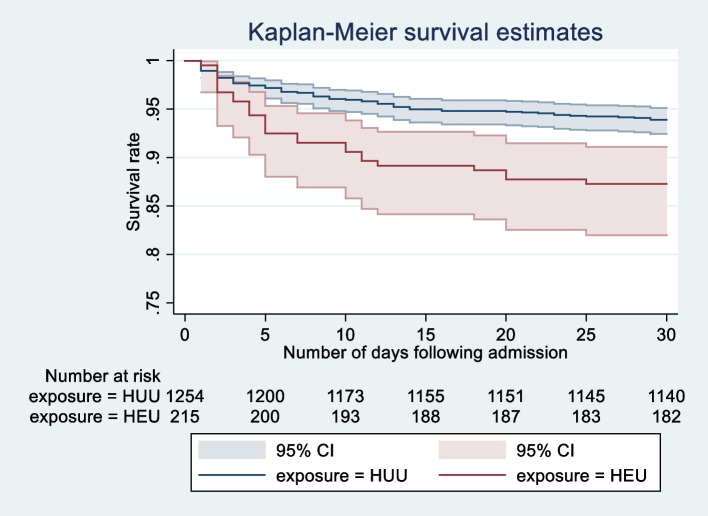
Kaplan–Meier survival curve showing survival rate versus number of days following hospital admission. Abbreviations: HUU HIV-unexposed uninfected, HEU HIV-exposed uninfected; logrank test for equality of survivor functions, *p* = 0.0004

Among HEU and HUU children, 24/217 (11.1%) and 77/1269 (6.1%) died, respectively, during hospitalization. Thirty days following admission, 29/217 (13.4%) HEU children and 90/1269 (7.1%) HUU children died. Time-to-inpatient death was not significantly different, with a median number of 3 days among HUU and 4 days among HEU children (*p* = 0.23). In both unadjusted and adjusted models, a significant effect of HIV exposure on inpatient and 30-day mortality was observed (Table 3; Additional file 1: Tables S2, S3, and Fig. S1). Although a significant interaction between wasting status and mortality was not observed (*p* = 0.46 and *p* = 0.24 for inpatient and 30-day mortality, respectively), an exploratory analysis was performed where wasting status was included in the adjusted models. Here, the association between HIV exposure and inpatient mortality was no longer significant [aOR 1.6, 95% CI (0.91–2.79; *p* = 0.10)], while the association between HIV exposure and 30-day mortality remained significant [aHR 1.85, 95% CI (1.01–3.36; *p* = 0.045)]. Survival curves by HIV exposure status are shown in Fig. 2.

**Table 3:** 30-day and inpatient mortality categorized by HIV exposure group

**30-day mortality**						
**Exposure**	**Unadjusted model**			**Adjusted model**		
	HR	CI	*p* value	HR	CI	*p* value
HUU	Ref	–	–	Ref	–	–
HEU	2.06	1.28–3.31	**0.003**	2.20	1.10–4.42	**0.027**
**Inpatient mortality**						
**Exposure**	**Unadjusted model**			**Adjusted model**		
	OR	CI	*p* value	OR	CI	*p* value
HUU	Ref	–	–	Ref	–	–
HEU	1.74	1.04–2.90	**0.034**	1.96	1.14–3.37	**0.014**

Abbreviations: *HUU* HIV-unexposed uninfected, *HEU* HIV-exposed uninfected, *HR* hazard ratios, *OR* odds ratio. Adjusted model included limited household assets, high food insecurity, recruitment site, prolonged travel time, age category (<6 months, 6–12 months, >12 months), and sex (see Additional file 1: Fig. S1 and Tables S2, S3)

### HIV exposure and nutritional status

As shown in the unadjusted analysis in Table 1, HIV exposure was associated with stunting but not wasting. However, in the multivariable-adjusted model, HEU children demonstrated nearly 50% higher odds of being wasted [aOR 1.41 (95% CI 1.03‒1.95)] and nearly a two-fold odds of being stunted at admission to hospital [aOR 1.91 (95% CI 1.34–2.70)] (Table 4; Additional file 1: Tables S4, S5, and Fig. S2).

**Table 4:** Summary of odds ratios of wasting and stunting at admission by HIV exposure

Wasting/stunting at admission						
Exposure	Unadjusted model			Adjusted model		
	Odds ratio	CI	*p* value	Odds ratio	CI	*p* value
HUU	Ref	–	–	Ref	–	–
HEU^a^	1.27	0.94–1.71	0.120	1.41	1.03–1.95	**0.034**
HEU^b^	1.75	1.27–2.41	**0.001**	1.91	1.34–2.70	**< 0.001**

*Abbreviations*: *HUU* HIV Unexposed uninfected, *HEU* HIV Exposed uninfected

^a^Odds ratio of wasting by HIV exposure category

^b^Odds ratio of stunting by HIV exposure category. Adjusted model included limited household assets, high food insecurity, recruitment site, prolonged travel time, age category (<6 months, 6–12 months, >12 months), and sex (see Additional file 1: Fig. S2 and Tables S4, S5)

### HIV exposure, duration of hospitalization, and use of inpatient resources

In the unadjusted model, the odds of having a prolonged (> 5 days) hospital stay among HEU children was not significantly different from HUU children (Table 5; Additional file 1: Fig. S3). However, in the multivariate model, the odds of prolonged hospitalization among HEU children was significantly higher than that observed for HUU children (Table 5; Additional file 1: Table S6 and Fig. S4). We also compared the use of inpatient resources (supplemental oxygen, nasogastric tube placement, and switch to second-line antibiotics) between HEU and HUU children. There were no significant differences in the requirement for use of inpatient resources between HEU and HUU children (Additional file 1: Tables S7–S10 and Fig. S5).

**Table 5:** Summary of odds ratios of having a prolonged hospitalization by HIV exposure

Prolonged length of hospital stay						
Exposure	Unadjusted model			Adjusted model		
	Odds ratio	CI	*p* value	Odds ratio	CI	*p* value
HUU	Ref	–	–	Ref	–	–
HEU	1.33	0.94–1.89	0.101	1.58	1.08–2.29	**0.017**

Adjusted model included limited household assets, high food insecurity, recruitment site, prolonged travel time, age category (<6 months, 6–12 months, >12 months), and sex (Additional file 1: Table S6 and Fig. S4)

*HEU* HIV Exposed uninfected, *HUU* HIV Unexposed uninfected

### HIV exposure, illness severity, admission diagnosis, and presence of clinical danger signs

We identified that age < 6 months was an effect modifier of high illness severity with a significant interaction with HIV exposure status (*p* = 0.013; Additional file 1: Table S11 and Fig. S6). However, our age-stratified and adjusted models did not identify significant associations between a high illness severity score and HIV exposure for children less than or greater than 6 months. We did observe that the association between illness severity and HIV exposure was different by age, however. Specifically, younger, HEU children were less likely to demonstrate high illness severity, whereas HEU children ≥ 6 months were more likely to demonstrate high illness severity (Additional file 1: Table S11). Admission diagnosis did not differ between HEU and HUU children (Table 1). There were no significant differences in the frequency and types of danger signs reported between HEU and HUU children (Additional file 1: Tables S12, S13, and Fig. S5).

## Discussion

The effective implementation of PMTCT programs to limit perinatal HIV transmission represents one of the most important public health interventions to promote early childhood survival, particularly in parts of sub-Saharan Africa where the prevalence of women-living-with-HIV remains high. Over the past two decades, however, HEU young children have emerged as an expanding vulnerable population that experiences increased mortality and morbidities, in both limited and well-resourced settings [8, 9, 11, 12, 27, 28]. This analysis explored associations between HIV-exposure-without-infection and inpatient and 30-day mortality, as well as multiple clinical and sociodemographic characteristics, to provide insight into drivers of poor outcomes among hospitalized HEU young children. HEU children were more likely to die during their hospitalization and in the immediate period following discharge (30-day mortality) [11]. HEU children were more likely to be wasted and stunted at hospital admission, and their households reported greater food insecurity and fewer economic resources. HEU children were more likely to require a prolonged hospital stay, although we did not detect significant differences in illness severity, admission diagnosis, the presence of clinical danger signs, or an increased requirement for inpatient resources among HEU children. Young, hospitalized, HEU children represent a distinct population with increased vulnerability to wasting and stunting, prolonged hospitalization, and death during and within 30 days of hospitalization.

Malnutrition, specifically severe wasting, is a well-recognized risk factor for early childhood mortality and morbidities and is likely to mediate the impact of HIV exposure on survival outcomes (Additional file 1: Fig. S7) [29, 30]. Here we determined that HEU children experienced significantly higher 30-day mortality rates and that their hazard of death remained elevated in analytic models both with and without adjustment for wasting. Thus, the increased 30-day mortality does not appear to be related to poor nutritional status alone in this cohort of HEU children. Together these findings support that HEU children under 2 years of age are uniquely vulnerable to dying during hospitalization and the early post-discharge period, regardless of nutritional status.

Although malnutrition has been associated with pediatric HIV infection [31], the association between malnutrition and HIV exposure among HEU children remains controversial [29–31]. HEU children may be more vulnerable to undernutrition due to the reduced prevalence of breastfeeding in this population (as observed here), as well as the economic effects of HIV infection on the caregiver and/or household that may limit access to age-appropriate complementary foods. In our study, households of HEU children were more likely to report high food insecurity and to have fewer household assets. However, we carefully assessed for confounding between HIV exposure and sociodemographic factors and subsequently adjusted for covariates including travel time to the hospital, high household food insecurity, and limited household assets. We hypothesize that feeding practices (particularly limited breastfeeding) were the primary driver of poor nutritional status among HEU children in this study [32]. We noted that HEU children were more likely to have ceased breastfeeding by 3 months of age, and less likely to be breastfeed beyond 12 months of age, as compared to HUU children. Additional biological factors that we were unable to quantify in our analysis, such as intestinal dysbiosis, metabolic derangements, and/or subclinical infections such as cytomegalovirus, could have also contributed to wasting [33–36] and the poor outcomes observed among HEU children (Additional file 1: Fig. S7).

Despite the increased inpatient and 30-day mortality observed here, there was no significant difference in illness severity at admission between HEU and HUU children. Our findings contrast with those reported in South Africa, where HEU children in the community had 4 times greater odds of experiencing a very severe infection when compared to HUU children [15]. However, differences in the definitions utilized to identify high illness severity between studies may have contributed to disparities reported across different studies. We did observe significantly higher odds of prolonged hospital stay among HEU children. Although prolonged hospitalization has been previously reported among HEU children [15], the drivers of prolonged stay remain unclear. We did not observe differences in age or prematurity between our HEU and HUU populations that could have contributed to the length of stay. The frequencies and types of danger signs also did not differ between HEU and HUU children. It is possible that the higher prevalence of wasting among HEU children in our study population masked typical clinical signs of sepsis, as has been reported among severely wasted HUU children [37]. We also did not detect significant differences in the need for hospital resources between HEU and HUU children. The presence of sub-clinical infection(s) or acquisition of nosocomial infection(s) may have been more likely to complicate recovery among HEU children and lead to prolonged hospital stay. Prolonged hospitalization not only places HEU children at increased risk for hospital-acquired infection and emergence of antimicrobial resistance but also increases the financial burden on the family. Our findings suggest that HEU children should be considered a high risk population during hospitalization and in the early post-discharge period, regardless of perceived illness severity and nutritional status at admission.

The study has several strengths. Firstly, this is a large multi-site study involving both rural and urban African sites making the findings generalizable across high-burden HIV settings in sub-Saharan Africa. Secondly, comprehensive and relevant data were systematically collected that encompassed child, caregiver, and household characteristics so that covariates could be considered in our statistical approaches. Thirdly, this is one of the few studies to examine both inpatient and 30-day mortality in young, African children. Finally, the study also included detailed data on nutritional status, illness severity, length-of-stay, and daily use of hospital resources, between HEU and HUU children. However, the study also had several limitations. One of the limitations of this analysis is that for the nutritional status and illness severity as outcomes, the design was cross-sectional, and therefore temporal relationships between variables cannot be ascertained. Secondly, maternal clinical and immunological variables that could affect study outcomes, such as the use of highly active antiretroviral therapy, CD4 count, and HIV viral load, were not collected in this study. Finally, as an observational study, we cannot assume that our findings are free from confounding despite our extensive efforts to control for these factors in our analysis.

Our findings have several implications for future research. Longitudinal studies, preferably beginning in pregnancy, are likely to be required to understand how perinatal HIV exposure in the absence of infection compromises early childhood health. Identifying biological drivers of increased hospital mortality, wasting, and prolonged hospitalization among HEU children is essential to develop clinical care guidelines tailored to support this unique and vulnerable population.

## Conclusions

Young children who are HIV-exposed but uninfected are more likely to present to hospital wasted and stunted. Following admission, young HEU children are at increased risk of death during their hospitalization and within 30 days of admission, and to require a prolonged hospitalization. These specific risks appear independent of social and demographic factors such as limited household assets and food security that are more prevalent among children with HIV exposure.

## Recommendations

Public health programs and hospital systems serving populations with a high prevalence of women-living-with-HIV need to ensure that HIV exposure status is established among HIV-uninfected children requiring hospitalization by testing both mothers and children. Hospitalized HEU young children should be managed as a population with increased vulnerability to death during hospitalization and within 30 days of admission, and be prioritized for additional support and post-discharge assessments to monitor recovery. Research that delineates the biologic mechanisms driving poor outcomes among HEU children is needed to develop effective strategies that improve survival and shape clinical care guidelines for this growing population of vulnerable children.

## Supplementary Information

Additional file 1: Supplemental materials. Supplemental methods. Supplemental data Tables S1–S13. Table S1 Age stopped breastfeeding among HEU vs HUU children not breastfeeding at hospital admission. Table S2 Associations between inpatient mortality, HIV exposure, and key biologic variables. Table S3 Associations between 30-day mortality, HIV exposure, and key biologic variables. Table S4 Associations between wasting, HIV exposure, and key biologic variables. Table S5 Associations between stunting, HIV exposure, and key biologic variables. Table S6 Associations between prolonged hospital stay, HIV exposure, and key biologic variables. Table S7 Summary of total number of days on oxygen, NGT use, and experience of any danger sign. Table S8 Number of days on oxygen. Table S9 Number of days with NGT. Table S10 Antibiotics switch from first-line to second-/third-line antibiotic among HEU vs HUU children. Table S11 Associations between illness severity, HIV exposure, and key biologic variables. Table S12 Number of days with danger signs. Table S13 Experience of specific danger signs. Supplemental Figures 1–7. Fig. S1Directed acyclic graph illustrating hypothesized relationships between various clinical and sociodemographic variables, HIV exposure, and 30 day and inpatient mortality. Fig. S2 Directed acyclic graph illustrating hypothesized relationships between various clinical and sociodemographic variables, HIV exposure, and child nutritional status. Fig. S3 Histogram of duration of hospitalization by HIV exposure category. Fig. S4 Directed acyclic graph illustrating hypothesized relationships between various clinical and sociodemographic variables, HIV exposure, and hospital length of stay. Fig. S5 Directed acyclic graph illustrating hypothesized relationships between various clinical and sociodemographic variables, HIV exposure, and use of hospital resources and presence of danger signs. Fig. S6 Directed acyclic graph illustrating hypothesized relationships between various clinical and sociodemographic variables, HIV exposure, and illness severity at enrollment. Fig. S7 Directed acyclic graph illustrating hypothesized relationships between HIV exposure and study outcomes.

## Data Availability

The CHAIN cohort data and analysis code are deposited and may be requested at: https://dataverse.harvard.edu/dataset.xhtml?persistentId=doi:10.7910/DVN/5H5X0P [38].

## Abbreviations

AIDS: Acquired immunodeficiency syndrome
ART: Antiretroviral therapy
CHAIN: Childhood Illness and Nutrition Network
CI: Confidence interval
CRF: Case report form
DNA: Deoxyribonucleic acid
HIV: Human immunodeficiency virus
IRB: Institutional review board
OR: Odds ratio
PCR: Polymerase chain reaction
PMTCT: Prevention of mother to child transmission
SIRS: Systemic inflammatory response syndrome
SSA: Sub-Saharan Africa
UNICEF: United Nations Children’s Fund
WHO: World Health Organization
