# Supplementary material for: Impact of HIV exposure without infection on hospital course and mortality among young children in sub-Saharan Africa: a multi-site cohort study
Source: BMC Med. 2024 Dec 3;22:573. doi: 10.1186/s12916-024-03790-5 (PMC11613948; doi:10.1186/s12916-024-03790-5)
Supplement: Supplementary file 1 — Additional file 1: Supplemental materials. Supplemental methods. Supplemental data Tables S1–S13. Table S1 Age stopped breastfeeding among HEU vs HUU children not breastfeeding at hospital admission. Table S2 Associations between inpatient mortality, HIV exposure, and key biologic variables. Table S3 Associations between 30-day mortality, HIV exposure, and key biologic variables. Table S4 Associations between wasting, HIV exposure, and key biologic variables. Table S5 Associations between stunting, HIV exposure, and key biologic variables. Table S6 Associations between prolonged hospital stay, HIV exposure, and key biologic variables. Table S7 Summary of total number of days on oxygen, NGT use, and experience of any danger sign. Table S8 Number of days on oxygen. Table S9 Number of days with NGT. Table S10 Antibiotics switch from first-line to second-/third-line antibiotic among HEU vs HUU children. Table S11 Associations between illness severity, HIV exposure, and key biologic variables. Table S12 Number of days with danger signs. Table S13 Experience of specific danger signs. Supplemental Figures 1–7. Fig. S1Directed acyclic graph illustrating hypothesized relationships between various clinical and sociodemographic variables, HIV exposure, and 30 day and inpatient mortality. Fig. S2 Directed acyclic graph illustrating hypothesized relationships between various clinical and sociodemographic variables, HIV exposure, and child nutritional status. Fig. S3 Histogram of duration of hospitalization by HIV exposure category. Fig. S4 Directed acyclic graph illustrating hypothesized relationships between various clinical and sociodemographic variables, HIV exposure, and hospital length of stay. Fig. S5 Directed acyclic graph illustrating hypothesized relationships between various clinical and sociodemographic variables, HIV exposure, and use of hospital resources and presence of danger signs. Fig. S6 Directed acyclic graph illustrating hypothesized relationships between var [file 12916_2024_3790_MOESM1_ESM.docx]

**Impact of HIV exposure without infection on hospital course and 30-day mortality among young children in sub-Saharan Africa: a multi-site cohort study**

**ADDITIONAL FILE 1**

**TABLE OF CONTENTS**

Contents

[**Supplementary Methods** 1](#_Toc181171200)

[**Ethics approval** 2](#_Toc181171201)

[**Data management** 2](#_Toc181171202)

[**Missing data** 3](#_Toc181171203)

[**Inverse sample weights** 3](#_Toc181171204)

[**Resource Utilization data** 4](#_Toc181171205)

[**Supplementary Tables** 5](#_Toc181171206)

[**Table S1: Age stopped breastfeeding among HEU vs HUU children not breastfeeding at hospital admission** 5](#_Toc181171207)

[**Table S2: Associations between inpatient mortality, HIV exposure, and key biologic variables** 6](#_Toc181171208)

[**Table S3: Associations between 30-day mortality, HIV exposure, and key biologic variables** 7](#_Toc181171209)

[**Table S4: Associations between wasting, HIV exposure, and key biologic variables** 8](#_Toc181171210)

[**Table S5: Associations between stunting, HIV exposure, and key biologic variables** 9](#_Toc181171211)

[**Table S6: Associations between prolonged hospital stay, HIV exposure, and key biologic variables** 10](#_Toc181171212)

[**Table S7: Summary of total number of days on Oxygen, NGT use, and experience of any danger sign** 11](#_Toc181171213)

[**Table S8: Number of days on Oxygen** 12](#_Toc181171214)

[**Table S9: Number of days with NGT** 13](#_Toc181171215)

[**Table S10: Antibiotics switch from first-line to second/third-line antibiotic among HEU vs HUU children.** 14](#_Toc181171216)

[**Table S11: Associations between illness severity, HIV exposure, and key biologic variables** 15](#_Toc181171217)

[**Table S12: Number of days with danger signs** 16](#_Toc181171218)

[**Table S13: Experience of specific danger signs** 17](#_Toc181171219)

[**Supplemental Figures** 18](#_Toc181171220)

# **Supplementary Methods**

## **Ethics approval**

The study was approved by Oxford Tropical Research Ethics Committee, the Scientific & Ethical Review Unit (SERU), Kenya Medical Research Institute in Kenya, Makerere University School of Biomedical Sciences Research Ethics Committee in Uganda, and COMREC, Kamuzu University of Health Sciences in Malawi.

## **Data management**

Details of data collection, data entry, and data cleaning are given in the CHAIN mortality paper (11)

**Statistical Approach**

All available data from hospitalized HEU and HUU children at the Ugandan, Kenyan, and Malawi CHAIN sites were included, and children documented to be living-with-HIV were excluded. *A priori* power calculations were not performed. The main exposure variable considered was perinatal HIV exposure, with analysis focusing on HEU and HUU children. The study outcomes were inpatient mortality, 30-day mortality, nutritional status at admission, illness severity at admission, hospital length of stay, presence of danger signs, and utilization of inpatient resources (supplemental oxygen, feeding tubes, and second-line antibiotics). All the analyses are weighted to reflect the stratification by nutritional status using inverse weights (1, 0.40, 0.39 for the not wasted, moderately wasted, and severely wasted and/or kwashiorkor) as explained elsewhere (11). Children’s characteristics at the time of admission to hospital stratified by HIV exposure category (HEU and HUU) are reported.

For all outcomes, directed acyclic graphs (DAGs) were developed to portray relationships between biologic, social, and demographic variables hypothesized to interact with each outcome and to delineate confounders, mediators, effect modifiers, and their relationships between the exposure variable and each outcome (Fig. S1-6). Variables selected for consideration included: age group (< 6 months, 6-11 months, and 12-24 months), biologic sex, breastfeeding status at enrollment, prematurity, nutritional wasting, limited household assets, high food insecurity, mother as primary caregiver, prolonged travel time to hospital, and recruitment site. Age < 6 months as tested as a potential effect modifier for each individual outcome (using a multiplicative interaction term between age and HIV exposure), and stratification performed if a significant interaction (p ≤0.05) was identified. Subsequent analytic models were developed for each outcome to support adjustment for confounding variables (26) as detailed below.

For inpatient mortality, days to death between the HIV exposure groups were compared using Wilcoxon Rank Sum test. To assess the effect of HIV exposure on inpatient mortality, a logistic regression model adjusting for sex, age group, limited household assets, high food insecurity, prolonged travel time to hospital, and recruitment site was used. Potential mediators, including duration of breastfeeding, prematurity, wasting, and mother as primary caregiver were not included in the final adjusted model so that the total impact of HIV-exposure on mortality could be observed (26) (Fig. S1 and Table S2). For 30-day mortality, multilevel parametric survival regression model with a Weibull probability distribution (as previously reported) (11) was performed. Adjustments were performed as described for inpatient mortality, with the exception that site adjustment was performed using a random-effects approach to be consistent with our prior work (11). Potential mediators, including breastfeeding status, prematurity, illness severity, and wasting status, were not included in the final adjusted models so that the total impact of HIV-exposure on each outcome could be observed (Fig. S1 and Table S3). We hypothesized that wasting status and/or age < 6 months could modify each outcome and formally tested for effect modification. Age < 6 months did not have a significant interaction with inpatient (p=0.9) or 30-day (p=0.87) mortality. Wasting status (yes/no) also did not have a significant interaction with inpatient (p=0.46) or 30-day (p=0.24) mortality. Despite not observing a significant interaction between wasting status and the mortality outcomes, an exploratory analysis that included adjustment for wasting status as a confounding variable for both inpatient and 30-day mortality was also performed, as reported in the Results section.

To assess the relationship between HIV exposure and nutritional status (binary wasted vs not wasted, and stunted vs not stunted) at hospital admission, logistic regression models adjusting for sex, age group, limited household assets, high food insecurity, prolonged travel time to hospital, and recruitment site were used. Potential mediators, including breastfeeding status, prematurity, and mother as primary caregiver, were not included in the final adjusted model so that the total impact of HIV exposure on nutritional status could be observed (Fig. S2 and Tables S4 and S5).

To assess the relationship between HIV exposure and illness severity (binary low and high), stratification based on age group (< 6 months versus > 6 months) was performed as age < 6 months was found to have a significant interaction with the outcome (p=0.013). Age-stratified logistic regression models adjusted for sex, limited household assets, high food insecurity, prolonged travel time to hospital, and recruitment site were used. Potential mediators, breastfeeding status, prematurity, mother as primary caregiver, and wasting were not included in the final adjusted model so that the total impact of HIV exposure on illness severity could be observed (Fig. S3 and Table S11).

To assess the relationship between HIV exposure and prolonged hospitalization, duration of hospitalization was categorized into binary variable using the median length of hospital stay (5 days) as the cut-off: short and prolonged duration of hospital stay. Length of hospital stay analysis was restricted to survivors and also excluded those who left against medical advice or absconded. We hypothesized that wasting status and/or age < 6 months could modify the length of hospitalization and formally tested for effect modification, with neither variable demonstrating a significant interaction p=0.1 and p=0.13, respectively). A logistic regression model adjusting for sex, age group, limited household assets, high food insecurity, prolonged travel time to hospital, and recruitment site were used. Potential mediators, including prematurity, illness severity, wasting status, and mother as primary caregiver, were not included in the final adjusted model so that the total impact of HIV exposure on prolonged hospital length of stay could be observed (Fig. S5 and Tables S6).

To assess the effect of HIV exposure on occurrence of daily danger signs and resource utilization (oxygen use, nasogastric tube use, and antibiotic switch), days with any danger sign or the use of a specific resource were counted. A zero-inflated negative binomial regression model was applied because the days with a danger sign or using any of the resources had leading zeros and was over-dispersed. The zero-inflated negative binomial regression was conducted for days with any danger sign and each resource separately. The reported measure of effect was incident rate ratio and corresponding 95% confidence intervals (95% CI). Potential mediators, including prematurity, illness severity, wasting status, and mother as primary caregiver, were not included in the final adjusted models so that the total impact of HIV exposure on each outcome could be observed (Fig. S4 and Tables S7 – S9, S12 and S13)

All statistical analyses were conducted using STATA College Station TX version 15.0 and the level of significance was assessed using two-tailed α <0.05 or 95% CIs.

## **Missing data**

Multiple imputation methods were used to cater for missing data. For continuous variables, the predicted measurements were estimated after a linear regression of the observed measurements with age, site, and sex, stratified by the 3 enrolment strata. Missing values were replaced with the mean predicted values in each group. For categorical (binary variables), the predicted probability was estimated after a logistic regression of each variable with age, site, and sex, stratified by the 3 enrolment strata; missing values were replaced by zero category (attribute not present) if the mean predicted values <0.5 and one category (attribute present) if the mean predicted values ≥0.5 in each group. Household variables with missing data were imputed using the iterative principal component analysis (PCA) method before running PCA on complete observations (11).

## **Inverse sample weights**

Because of the non-proportional stratified sampling in the original CHAIN study, we created sampling weights (3 weights for the 3 strata) proportional to the inverse of the sampling fraction of the respective group from a typical hospital admission in Africa and South Asia. Hospital pediatric admission surveillance data (for children 2 to 23 months old) during the period of the CHAIN study from four site hospitals were used to estimate the mean proportions across the three nutrition strata. Using the estimated proportions and the actual proportion of children recruited in the CHAIN study, we calculated the inverse probability of children being recruited in each nutrition stratum and standardized the inverse probability by dividing with the NW group probability. This resulted in 3 weights: 1.0, 0.40, and 0.39, for the not wasted, moderately wasted, and severely wasted or kwashiorkor groups respectively (11).

**Resource Utilization data**:

**Data collection**

Data on participants’ daily clinical events, clinical observations, and medications were captured on the daily record CRF. These included the experience of WHO danger signs in the last 24 hours (obstructed breathing, cyanosis, respiratory distress, shock, severe anemia, convulsions, severe dehydration, profuse watery diarrhea, vomiting everything, and impaired consciousness). Further, data on oxygen use, nasogastric use, antibiotic use (crystalline/benzylpenicillin, gentamicin, ceftriaxone, ampicillin, amikacin, ciprofloxacin, co-amoxiclav, chloramphenicol, cefotaxime, ceftazidime, flucloxacillin/cloxacillin, meropenem/imipenem, levofloxacin) were collected.

**Analysis of Use of Hospital Resources & Presence of Danger Signs**

**Oxygen use**

We determined which participants had at least one day of oxygen use and computed the total number of days on oxygen by HIV exposure group. We conducted zero-inflated negative binomial regression to detect differences in the use of oxygen between the two HIV exposure groups (HEU and HUU) and reported the incident rate ratio and the corresponding p-value. Sex, age, limited household assets, high food insecurity, recruitment site, and prolonged travel time were included in the count model. Sex, age, site, illness severity and nutritional status at admission were put in the inflation model.

**Nasogastric tube use**

We determined which participants had at least one day of nasogastric tube use and computed the total number of days during which a nasogastric tube was used by HIV exposure group. We used zero-inflated negative binomial regression to detect differences in the use of the nasogastric tube between the two HIV exposure groups (HEU and HUU) and reported the incident rate ratio and the corresponding p-value. Sex, age, limited household assets, high food insecurity, recruitment site, and prolonged travel time were included in the count model. Sex, age, site, illness severity, and nutritional status at admission were put in the inflation model.

**Occurrence of danger signs**

We determined which participants had at least one day of experiencing danger signs and the total number of days with at least a danger sign by HIV exposure groups (HEU and HUU). We used zero-inflated negative binomial regression to detect differences in the number of days with danger signs between the two HIV exposure groups (HEU and HUU). Sex, age, limited household assets, high food insecurity, recruitment site, and prolonged travel time were included in the count model. We reported the incident rate ratio, 95% confidence interval, and corresponding p-value.

**Switching of antibiotics**

We defined switching of antibiotics as changing from the first-line medication (intravenous ampicillin/benzylpenicillin and gentamicin) to a second-line or third-line intravenous antibiotic (cephalosporin, fluoroquinolone, carbapenem, and amphenicols). We compared the occurrence of antibiotic switch between the HIV exposure groups (HEU and HUU) using the chi-square test and reported the p-value.

# **Supplementary Tables**

## **Table S1: Age stopped breastfeeding among HEU vs HUU children not breastfeeding at hospital admission**

| **Factor** | **HUU** | **HEU** | **Total** | **P-value*** |
| --- | --- | --- | --- | --- |
| **Age** | |  |  |  |
| 0 - 3 months | 78 (20.91) | 32 (28.32) | 110 (22.63) | 0.011 |
| 4 -6 months | 47 (12.60) | 18 (15.93) | 65 (13.37) |  |
| 7 - 12 months | 113 (30.30) | 34 (30.09) | 147 (30.25) |  |
| > 12 months | 110 (29.49) | 18 (15.93) | 128 (26.34) |  |
| Unknown | 25 (6.70) | 11 (9.73) | 36 (7.41) |  |
| Total | 373 | 113 | 486 |  |

*chi-square test

## **Table S2: Associations between inpatient mortality, HIV exposure, and key biologic variables**

| **Factors** | **Crude OR**  **(95% CI)** | **P-value** | **Adjusted OR (95% CI)** | **P-value** |
| --- | --- | --- | --- | --- |
| **HIV exposure** |  |  |  |  |
| HUU | Ref | - | Ref | - |
| HEU | 1.74 (1.04-2.90) | 0.034 | 1.96 (1.14-3.37) | 0.014 |
| **Sex** |  |  |  |  |
| Male | Ref | - | Ref | - |
| Female | 1.11 (0.72-1.72) | 0.641 | 1.14 (0.70-1.86) | 0.595 |
| **Hospital travel time** | |  |  |  |
| < 1 hour | Ref | - | Ref | - |
| 1 – 2 hours | 0.97 (0.58-1.64) | 0.918 | 0.91 (0.54-1.54) | 0.735 |
| >2 hours | 1.30 (0.65-2.63) | 0.459 | 1.34 (0.61-2.95) | 0.463 |
| **Age group** |  |  |  |  |
| 12 and above | Ref | - | Ref | - |
| 6 to 11 | 1.81 (1.13-2.92) | 0.014 | 1.75 (1.02-2.99) | 0.041 |
| <6 months | 1.32 (0.69-2.55) | 0.401 | 1.30 (0.63-2.69) | 0.484 |
| **Food insecurity** |  |  |  |  |
| Low | Ref | - | Ref | - |
| Moderate | 0.59 (0.35-0.98) | 0.041 | 0.73 (0.41-1.30) | 0.278 |
| High | 1.19 (0.71-2.02) | 0.509 | 1.37 (0.73-2.56) | 0.332 |
| **Household assets** |  |  |  |  |
| Poorest | Ref | - | Ref | - |
| Second | 0.57 (0.29-1.12) | 0.105 | 0.81 (0.37-1.79) | 0.608 |
| Middle | 1.02 (0.59-1.74) | 0.955 | 0.84 (0.37-1.88) | 0.670 |
| Fourth | 0.30 (0.14-0.65) | 0.002 | 0.46 (0.18-1.15) | 0.097 |
| Least poor | 0.28 (0.10-0.73) | 0.009 | 0.51 (0.15-1.70) | 0.273 |
| **Site** |  |  |  |  |
| Kilifi | Ref | - | Ref | - |
| Mbagathi | 1.90 (0.75-4.78) | 0.173 | 1.89 (0.49-7.30) | 0.355 |
| Migori | 3.98 (1.68-9.43) | 0.002 | 5.24 (1.96-14.05) | 0.001 |
| Kampala | 1.23 (0.49-3.06) | 0.658 | 1.87 (0.54-6.51) | 0.324 |
| Blantyre | 0.80 (0.30-2.18) | 0.669 | 1.14 (0.34-3.83) | 0.835 |

Abbreviations: OR-Odds ratios, CI-Confidence intervals.
Adjusted model included: sex, age, limited household assets, high food insecurity, recruitment site, and prolonged travel time (see Fig. S1).

## **Table S3: Associations between 30-day mortality, HIV exposure, and key biologic variables**

| **Factors** | **Crude HR ( 95% CI)** | **P-value** | **Adjusted HR (95% CI)** | **P-value** |
| --- | --- | --- | --- | --- |
| **HIV exposure** |  |  |  |  |
| HUU | Ref | - | Ref | - |
| HEU | 2.06 (1.28-3.31) | 0.003 | 2.20 (1.10-4.42) | 0.027 |
| **Sex** |  |  |  |  |
| Male | Ref | - | Ref | - |
| Female | 1.01 (0.67-1.55) | 0.949 | 1.04 (0.81-1.33) | 0.762 |
| **Hospital travel time** | |  |  |  |
| < 1 hour | Ref | - | Ref | - |
| 1 – 2 hours | 1.01 (0.62-1.66) | 0.954 | 0.98 (0.75-3.24) | 0.907 |
| >2 hours | 1.21 (0.61-2.37) | 0.585 | 1.15 (0.51-2.63) | 0.732 |
| **Age group** |  |  |  |  |
| 12 and above | Ref | - | Ref | - |
| 6 to 11 | 1.48 (0.93-2.33) | 0.093 | 1.56 (0.75-3.24) | 0.233 |
| <6 months | 1.35 (0.71-2.55) | 0.355 | 1.46 (0.59-3.58) | 0.41 |
| **Food insecurity** |  |  |  |  |
| Low | Ref | - | Ref | - |
| Moderate | 0.78 (0.47-1.28) | 0.327 | 0.84 (0.67-1.06) | 0.139 |
| High | 1.41 (0.85-2.35) | 0.184 | 1.36 (0.95-1.96) | 0.098 |
| **Household assets** |  |  |  |  |
| Poorest | Ref | - | Ref | - |
| Second | 0.52 (0.26-1.02) | 0.058 | 0.57 (0.16-1.97) | 0.372 |
| Middle | 1.04 (0.61-1.77) | 0.871 | 0.88 (0.33-2.33) | 0.797 |
| Fourth | 0.46 (0.24-0.90) | 0.023 | 0.51 (0.17-1.58) | 0.245 |
| Least poor | 0.36 (0.51-0.88) | 0.026 | 0.47 (0.09-2.29) | 0.347 |

Abbreviations: HR-Hazard ratios, CI-Confidence intervals.

Adjusted model included: sex, age, limited household assets, high food insecurity, recruitment site, and prolonged travel time (see Fig. S1).

## **Table S4: Associations between wasting, HIV exposure, and key biologic variables**

| **Factors** | **Crude OR (95% CI)** | **P-value** | **Adjusted OR (95% CI)** | **P-value** |
| --- | --- | --- | --- | --- |
| **HIV Exposure** |  |  |  |  |
| HUU | Ref | - | Ref | - |
| HEU | 1.27 (0.94-1.71) | 0.119 | 1.41 (1.03-1.95) | 0.034 |
| **Sex** |  |  |  |  |
| Male | Ref | - | Ref | - |
| Female | 1.29 (1.05-1.59) | 0.016 | 1.35 (1.08-1.68) | 0.009 |
| **Hospital travel time** |  |  |  |  |
| < 1 hour | Ref | - | Ref | - |
| 1-2 hours | 1.28 (1.02-1.61) | 0.03 | 1.20 (0.94-1.53) | 0.145 |
| > 2 hours | 2.22 (1.57-3.16) | <0.001 | 1.86 (1.28-2.70) | 0.001 |
| **Age group** |  |  |  |  |
| 12 and above | Ref | - | Ref | - |
| 6 – 11 months | 1.09 (0.86-1.37) | 0.472 | 1.05 (0.82-1.34) | 0.724 |
| < 6 months | 0.93 (0.69-1.24) | 0.618 | 0.95 (0.70-1.30) | 0.76 |
| **Food insecurity** |  |  |  |  |
| Low | Ref | - | Ref | - |
| Moderate | 1.26 (1.0-1.59) | 0.047 | 1.13 (0.89-1.45) | 0.318 |
| High | 2.13 (1.57-2.89) | <0.001 | 2.08 (1.49-2.91) | <0.001 |
| **Household assets** |  |  |  |  |
| Poorest | Ref | - | Ref | - |
| Second | 0.98 (0.71-1.34) | 0.885 | 0.96 (0.68-1.36) | 0.83 |
| Middle | 1.29 (0.96-1.74) | 0.088 | 0.85 (0.58-1.25) | 0.405 |
| Fourth | 1.02 (0.75-1.38) | 0.892 | 0.64 (0.42-0.96) | 0.032 |
| Least poor | 1.24 (0.84-1.82) | 0.284 | 1.04 (0.64-1.68) | 0.882 |
| **Site** |  |  |  |  |
| Kilifi | Ref | - | Ref | - |
| Mbagathi | 2.71 (1.51-3.12) | <0.001 | 2.24 (1.43-3.49) | <0.001 |
| Migori | 1.55 (1.08-2.23) | 0.017 | 1.46 (1.0-2.14) | 0.05 |
| Kampala | 2.65 (1.9-3.68) | <0.001 | 3.06 (2.08-4.49) | <0.001 |
| Blantyre | 0.75 (0.53-1.07) | 0.113 | 0.70 (0.48-1.02) | 0.063 |

Abbreviations: OR-Odds ratios, CI-Confidence intervals.

Adjusted model included: sex, age, limited household assets, high food insecurity, recruitment site, and prolonged travel time (see Fig. S2).

## **Table S5: Associations between stunting, HIV exposure, and key biologic variables**

| **Factors** | **Crude OR (95% CI)** | **P-value** | **Adjusted OR (95% CI)** | **P-value** |
| --- | --- | --- | --- | --- |
| **HIV Exposure** |  |  |  |  |
| HUU | Ref | - | Ref | - |
| HEU | 1.75 (1.27-2.41) | 0.001 | 1.91 (1.34-2.70) | <0.001 |
| **Sex** |  |  |  |  |
| Male | Ref | - | Ref | - |
| Female | 0.72 (0.57-0.90) | 0.003 | 0.68 (0.54-0.87) | 0.002 |
| **Hospital travel time** |  |  |  |  |
| < 1 hour | Ref | - | Ref | - |
| 1-2 hours | 1.31 (1.02-1.67) | 0.031 | 1.33 (1.02-1.73) | 0.031 |
| > 2 hours | 1.43 (1.0-2.03) | 0.045 | 1.23 (0.83-1.80) | 0.299 |
| **Age** |  |  |  |  |
| >=12 months | Ref | - | Ref | - |
| 6-11 months | 0.59 (0.46-0.76) | <0.001 | 0.56 (0.43-0.73) | <0.001 |
| < 6 months | 0.53 (0.38-0.73) | <0.001 | 0.53 (0.37-0.75) | <0.001 |
| **Food insecurity** |  |  |  |  |
| Low | Ref | - | Ref | - |
| Moderate | 1.14 (0.89-1.47) | 0.285 | 0.95 (0.72-1.25) | 0.732 |
| High | 2.07 (1.51-2.83) | <0.001 | 1.80 (1.27-2.53) | 0.001 |
| **Household assets** |  |  |  |  |
| Poorest | Ref | - | Ref | - |
| Second | 0.93 (0.66-1.31) | 0.688 | 0.82 (0.55-1.20) | 0.303 |
| Middle | 1.12 (0.82-1.54) | 0.482 | 0.77 (0.52-1.15) | 0.2 |
| Fourth | 0.88 (0.63-1.22) | 0.429 | 0.65 (0.42-1.02) | 0.058 |
| Least poor | 0.79 (0.52-1.20) | 0.279 | 0.69 (0.41-1.17) | 0.166 |
| **Site** |  |  |  |  |
| Kilifi | Ref | - | Ref | - |
| Mbagathi | 1.51 (1.02-2.24) | 0.04 | 1.77 (1.08-2.87) | 0.022 |
| Migori | 1.43 (0.96-2.14) | 0.078 | 1.26 (0.82-1.93) | 0.285 |
| Kampala | 2.65 (1.85-3.80) | <0.001 | 3.26 (2.13-4.98) | <0.001 |
| Blantyre | 1.50 (1.02-2.21) | 0.038 | 1.39 (0.92-2.12) | 0.122 |

Abbreviations: OR-Odds ratios, CI-Confidence intervals.

Adjusted model included: sex, age, limited household assets, high food insecurity, recruitment site, and prolonged travel time (see Fig. S2).

## **Table S6: Associations between prolonged hospital stay, HIV exposure, and key biologic variables**

| **Factors** | **Crude OR (95% CI)** | **P-value** | **Adjusted OR (95% CI)** | **P-value** |
| --- | --- | --- | --- | --- |
| **HIV Exposure** |  |  |  |  |
| HUU | Ref | - | Ref | - |
| HEU | 1.33 (0.94-1.89) | 0.101 | 1.58 (1.08-2.29) | 0.017 |
| **Sex** |  |  |  |  |
| Male | Ref | - | Ref | - |
| Female | 0.94 (0.74-1.19) | 0.593 | 0.98 (0.75-1.26) | 0.851 |
| **Hospital travel time** | |  |  |  |
| < 1 hour | Ref | - | Ref | - |
| 1 – 2 hours | 1.43 (1.10-1.85) | 0.007 | 1.22 (0.92-1.62) | 0.161 |
| >2 hours | 3.78 (2.57-5.56) | <0.001 | 2.88 (1.94-4.29) | <0.001 |
| **Age** |  |  |  |  |
| 12 and above | Ref | - | Ref | - |
| 6 to 11 | 0.86 (0.67-1.12) | 0.276 | 0.81 (0.61-1.08) | 0.159 |
| <6 months | 0.87 (0.62-1.23) | 0.434 | 0.86 (0.59-1.27) | 0.46 |
| **Food insecurity** | |  |  |  |
| Low | Ref | - | Ref | - |
| Moderate | 1.24 (0.95-1.65) | 0.106 | 1.01 (0.75-1.34) | 0.971 |
| High | 1.67 (1.20-2.33) | 0.002 | 1.69 (1.18-2.44) | 0.005 |
| **Household assets** | |  |  |  |
| Poorest | Ref | - | Ref | - |
| Second | 1.32 (0.91-1.91) | 0.144 | 1.23 (0.81-1.87) | 0.342 |
| Middle | 1.72 (1.21-2.42) | 0.002 | 0.96 (0.61-1.51) | 0.852 |
| Fourth | 1.82 (1.28-2.59) | 0.001 | 0.79 (0.47-1.34) | 0.381 |
| Least poor | 1.72 (1.10-2.67) | 0.015 | 0.89 (0.49-1.63) | 0.711 |
| **Site** |  |  |  |  |
| Kilifi | Ref | - | Ref | - |
| Mbagathi | 5.51 (3.59-8.4) | <0.001 | 6.23 (3.59-10.8) | <0.001 |
| Migori | 1.40 (0.91-2.18) | 0.130 | 1.41 (0.87-2.26) | 0.162 |
| Kampala | 2.50 (1.71-3.65) | <0.001 | 2.86 (1.78-4.60) | <0.001 |
| Blantyre | 0.63 (0.40-0.98) | p=0.04 | 0.58 (0.36-0.93) | 0.025 |

Abbreviations: OR-odds ratios.
Adjusted model included: sex, age, limited household assets, high food insecurity, recruitment site, and prolonged travel time (see Fig. S4).

## **Table S7: Summary of total number of days on Oxygen, NGT use, and experience of any danger sign**

| **Resource used/experience of any danger sign** | **No** | **Yes** | **Total days** | **Adjusted IRR (95% CI)** | **P value*** |
| --- | --- | --- | --- | --- | --- |
| **Oxygen use** |  |  |  |  |  |
| HUU | 987 (83.7) | 192 (16.3) | 710 | 0.90 (0.52-1.55) | 0.71 |
| HEU | 163 (84.5) | 30 (15.5) | 100 |  |  |
| **Nasogastric tube use** |  |  |  |  |  |
| HUU | 1053 (89.3) | 126 (10.6) | 471 | 0.79 (0.40-1.69) | 0.541 |
| HEU | 175 (90.7) | 18 (9.3) | 64 |  |  |
| **Any danger sign** |  |  |  |  |  |
| HUU | 941 (79.8) | 238 (20.2) | 695 | 0.73 (0.51-1.06) | 0.103 |
| HEU | 156 (80.8) | 37 (19.2) | 73 |  |  |

Abbreviations: IRR-Incident rate ratios, CI-Confidence intervals.

*Used zero-inflated negative binomial regression: sex, age, limited household assets, high food insecurity, recruitment site, and prolonged travel time (see Fig. S5) included in the adjusted count model; sex, age, size, nutritional status, and illness severity were put in the inflation model.

## **Table S8: Number of days on Oxygen**

| **Factor** | **Un adjusted IRR(95% CI)** | **p-value** | **Adjusted IRR(95% CI)** | **p-value*** |
| --- | --- | --- | --- | --- |
| **HIV Exposure** |  |  |  |  |
| HUU | Ref | - | Ref | - |
| HEU | 0.83 (0.40-1.70) | 0.604 | 0.90 (0.52-1.55) | 0.71 |
| **Sex** |  |  |  |  |
| Male | Ref | - | Ref | - |
| Female | 1.19 (0.72-1.96) | 0.494 | 1.05 (0.49-2.26) | 0.12 |
| **Hospital travel time** | |  |  |  |
| < 1 hour | Ref | - | Ref | - |
| 1 – 2 hours | 1.34 (0.78-2.30) | 0.286 | 0.81 (0.57-1.16) | 0.246 |
| >2 hours | 1.13 (0.59-2.14) | 0.720 | 0.81 (0.51-1.30) | 0.39 |
| **Age** |  |  |  |  |
| >=12 months | Ref | - | Ref | - |
| 6 to 11 | 2.51 (1.45-4.34) | 0.001 | 2.38 (1.39-4.08) | 0.002 |
| <6months | 2.78 (1.51-5.09) | 0.001 | 2.65 (1.50-4.66 | 0.001 |
| **Food insecurity** |  |  |  |  |
| Low | Ref | - | Ref | - |
| Moderate | 1.20 (0.69-2.09) | 0.514 | 1.26 (0.85-1.88) | 0.246 |
| High | 1.18 (0.60-2.33) | 0.627 | 1.33 (0.76-2.33) | 0.316 |
| **Household assets** |  |  |  |  |
| Poorest | Ref | - | Ref | - |
| Second | 0.69 (0.31-1.54) | 0.365 | 0.79 (0.37-1.67) | 0.532 |
| Middle | 0.71 (0.35-1.42) | 0.328 | 0.66 (0.36-1.19) | 0.169 |
| Fourth | 2.17 (1.09-4.31) | 0.027 | 0.99 (0.42-2.31) | 0.981 |
| Least poor | 1.56 (0.70-3.46) | 0.273 | 0.75 (0.28-2.02) | 0.571 |
| **Site** |  |  |  |  |
| Kilifi | Ref | - | Ref | - |
| Mbagathi | 1.77 (0.61-5.14) | 0.292 | 1.75 (0.39-7.85) | 0.467 |
| Migori | 0.69 (0.22-2.22) | 0.539 | 0.43 (0.05-4.14) | 0.728 |
| Kampala | 0.63 (0.20-1.95) | 0.423 | 0.84 (0.11-6.72) | 0.87 |
| Blantyre | 1.08 (0.34-3.45) | 0.902 | 0.67 (0.09-4.79) | 0.689 |

Abbreviations: IRR-Incident rate ratios, CI-Confidence intervals.

*Used zero-inflated negative binomial regression: sex, age, limited household assets, high food insecurity, recruitment site, and prolonged travel time (see Fig. S5) included in the adjusted count model; sex, age, size, nutritional status, and illness severity were included in the inflation model. Age < 6 months was tested as an effect modifier and not found to have a significant interaction (p=0.22)

## **Table S9: Number of days with NGT**

| **Factor** | **Un adjusted IRR(95% CI)** | **p-value** | **Adjusted IRR(95% CI)** | **p-value** |
| --- | --- | --- | --- | --- |
| **HIV Exposure** |  |  |  |  |
| HUU | Ref | - | Ref | - |
| HEU | 0.93 (0.44-1.98) | 0.844 | 0.79 (0.40-1.69) | 0.541 |
| **Sex** |  |  |  |  |
| Male | Ref | - | Ref | - |
| Female | 1.22 (0.74-2.0) | 0.440 | 0.99 (0.53-1.86) | 0.983 |
| **Hospital travel time** | |  |  |  |
| >=12 months | Ref | - | Ref | - |
| 6 to 11 | 0.97 (0.56-1.67) | 0.90 | 0.52 (0.32-0.82) | 0.006 |
| <6months | 1.39 (0.73-2.66) | 0.320 | 0.90 (0.46-1.76) | 0.753 |
| **Age** |  |  |  |  |
| >=12 months | Ref | - | Ref | - |
| 6 to 11 | 1.38 (0.81-2.37) | 0.238 | 0.79 (0.43-1.44) | 0.439 |
| <6months | 1.37 (0.64-2.91) | 0.418 | 0.42 (0.21-0.88) | 0.02 |
| **Food insecurity** |  |  |  |  |
| Low | Ref | - | Ref | - |
| Moderate | 1.24 (0.72-2.14) | 0.439 | 0.83 (0.50-1.38) | 0.48 |
| High | 1.50 (0.76-2.98) | 0.240 | 1.38 (0.70-2.70) | 0.352 |
| **Household assets** |  |  |  |  |
| Poorest | Ref | - | Ref | - |
| Second | 0.72 (0.32-1.60) | 0.416 | 0.69 (0.27-1.80) | 0.449 |
| Middle | 0.35 (0.17-0.73) | 0.005 | 0.44 (0.18-1.10) | 0.08 |
| Fourth | 0.64 (0.31-1.33) | 0.231 | 0.48 (0.19-1.25) | 0.132 |
| Least poor | 0.31 (0.12-0.81) | 0.017 | 0.22 (0.07-0.76) | 0.016 |
| **Site** |  |  |  |  |
| Kilifi | Ref |  | Ref |  |
| Mbagathi | 0.46 (0.18-1.16) | 0.099 | 1.85 (0.24-14.1) | 0.554 |
| Migori | 0.46 (0.13-1.63) | 0.231 | 2.27 (0.25-20.6) | 0.466 |
| Kampala | 0.59 (0.25-1.44) | 0.250 | 1.45 (0.28-7.53) | 0.656 |
| Blantyre | 0.36 (0.13-1.03) | 0.056 | 0.56 (0.11-2.90) | 0.492 |

Abbreviations: IRR-Incident rate ratios, CI-Confidence intervals.

*Used zero-inflated negative binomial regression: sex, age, limited household assets, high food insecurity, recruitment site, and prolonged travel time (see Fig. S5) included in the adjusted count model; sex, age, size, nutritional status, and illness severity were included in the inflation model. Age < 6 months was tested as an effect modifier and not found to have a significant interaction (p=0.42)

## **Table S10: Antibiotics switch from first-line to second/third-line antibiotic among HEU vs HUU children.**

|  | **Switched antibiotics** | |  |  |
| --- | --- | --- | --- | --- |
| **Exposure** | No | Yes | Total | p-value* |
| HUU | 844 (71.6) | 335 (28.4) | 1179 (100) | 0.188 |
| HEU | 147 (76.2) | 46 (23.8) | 193 (100) | |
| Total | 991 (72.2) | 381 (27.8) | 1372 (100) | |

*****Used chi-square test, OR= 0.78 (0.55-1.12, p = 0.189)

## **Table S11: Associations between illness severity, HIV exposure, and key biologic variables**

| **Factors** | **Crude OR(95% CI)** | **Adjusted OR (95% CI)**  **≥6 months** | **Adjusted OR (95% CI)**  **<6 months** |
| --- | --- | --- | --- |
| **HIV Exposure** |  |  |  |
| HUU | Ref | Ref | Ref |
| HEU | 1.02 (0.74-1.18)^a^ | 1.40 (0.96-2.04)^b^ | 0.47 (0.20-1.09)^c^ |
| **Sex** |  |  |  |
| Male | Ref | Ref | Ref |
| Female | 0.97 (0.77-1.23) | 1.03 (0.79-1.34) | 0.73 (0.40-1.34) |
| **Hospital travel time** | |  |  |
| <1 hour | Ref | Ref | Ref |
| 1-2 hours | 0.81 (0.63-1.05) | 0.73 (0.54-0.98)* | 0.96 (0.52-1.80) |
| >=2 hours | 0.93 (0.64-1.34) | 0.90 (0.59-1.38) |  |
| **Food insecurity** |  |  |  |
| Low | Ref | Ref | Ref |
| Moderate | 0.86 (0.66-1.11) | 0.99 (0.74-1.34) | 0.53 (0.26-1.05) |
| High | 0.92 (0.67-1.27) | 0.97 (0.65-1.44) | 0.62 (0.29-1.32) |
| **Household assets** |  |  |  |
| Poorest | Ref | Ref | Ref |
| Second | 0.56 (0.39-0.8)** | 0.63 (0.40-0.99)* | 0.38 (0.16-0.92)* |
| Middle | 0.81 (0.58-1.12) | 1.14 (0.73-1.76) | 0.31 (0.12-0.83)* |
| Fourth | 0.90 (0.65-1.26) | 1.17 (0.73-1.88) | 0.78 (0.30-2.04) |
| Least poor | 0.71 (0.47-1.09) | 0.98 (0.56-1.73) | 0.42 (0.12-1.42) |
| **Site** |  |  |  |
| Kilifi | Ref | Ref | Ref |
| Mbagathi | 0.83 (0.56-1.22) | 0.62 (0.37-1.04) | 1.71 (0.62-4.77) |
| Migori | 0.99 (0.67-1.48) | 0.96 (0.60-1.52) | 1.03 (0.43-2.43) |
| Kampala | 0.60 (0.42-0.86)** | 0.51 (0.33-0.8)** | 1.01 (0.35-2.90) |
| Blantyre | 0.63 (0.43-0.93)* | 0.58 (0.37-0.9)* | 1.29 (0.48-3.45) |

* p<0.05; ** p<0.01; *** p<0.001

Abbreviations: OR-Odds ratios, CI-Confidence intervals

Age < 6 months was tested as an effect modifier and found to have a significant interaction (p=0.013) with HIV-exposure status. A stratified analysis was subsequent performed with adjustment for sex, prolonged travel time to hospital, high food insecurity, limited household assets and recruitment site, among children ≥6 months and <6 months (Fig. S6).

## **Table S12: Number of days with danger signs**

| **Factor** | **Un adjusted IRR (95% CI)** | **p-value** | **Adjusted IRR (95% CI)** | **p-value*** |
| --- | --- | --- | --- | --- |
| **HIV Exposure** |  |  |  |  |
| HUU | Ref |  | Ref |  |
| HEU | 0.47 (0.26-0.85) | 0.012 | 0.73 (0.51-1.06) | 0.103 |
| **Sex** |  |  |  |  |
| Male | Ref |  | Ref |  |
| Female | 1.12 (0.73-1.71) | 0.601 | 1.11 (0.74-1.64) | 0.619 |
| **Hospital travel time** | |  |  |  |
| <1 hour | Ref | - | Ref | - |
| 1-2 hours | 1.04 (0.65-1.65) | 0.874 | 0.91 (0.63-1.30) | 0.596 |
| >=2 hours | 1.24 (0.71-2.17) | 0.446 | 1.29 (0.85-1.95) | 0.23 |
| **Age** |  |  |  |  |
| >=12 months | Ref | - | Ref | - |
| 6 to 11 | 1.57 (1.0-2.47) | 0.05 | 1.62 (1.05-2.49) | 0.028 |
| <6months | 0.76 (0.43-1.36) | 0.359 | 1.35 (0.75-2.43) | 0.312 |
| **Food insecurity** |  |  |  |  |
| Low | Ref | - | Ref | - |
| Moderate | 0.95 (0.59-1.52) | 0.831 | 1.01 (0.71-1.43) | 0.958 |
| High | 0.85 (0.48-1.53) | 0.595 | 1.01 (0.67-1.52) | 0.977 |
| **Household assets** |  |  |  |  |
| Poorest | Ref | - | Ref | - |
| Second | 1.18 (0.56-2.47) | 0.661 | 0.82 (0.42-1.63) | 0.581 |
| Middle | 0.62 (0.34-1.14) | 0.126 | 0.78 (0.43-1.45) | 0.437 |
| Fourth | 0.82 (0.46-1.48) | 0.511 | 0.82 (0.44-1.55) | 0.542 |
| Least poor | 0.59 (0.29-1.21) | 0.153 | 0.71 (0.32-1.59) | 0.407 |
| **Site** |  |  |  |  |
| Kilifi | Ref |  | Ref |  |
| Mbagathi | 0.49 (0.31-0.80) | 0.005 | 0.55 (0.26-1.16) | 0.117 |
| Migori | 0.33 (0.13-0.80) | 0.014 | 0.36 (0.13-1.02) | 0.054 |
| Kampala | 0.77 (0.45-1.31) | 0.333 | 0.87 (0.47-1.63) | 0.671 |
| Blantyre | 0.22 (0.10-0.47) | <0.001 | 0.22 (0.08-0.56) | 0.002 |

Abbreviations: IRR-Incident rate ratios, CI-Confidence intervals.

*Used zero-inflated negative binomial regression: sex, age, limited household assets, high food insecurity, recruitment site, and prolonged travel time (see Fig. S5) included in the adjusted count model; sex, age, size, nutritional status, and illness severity were put in the inflation model.

## **Table S13: Experience of specific danger signs**

|  | **Experience of specific danger signs** | |  |  |
| --- | --- | --- | --- | --- |
| **HIV Exposure** | **No** | **Yes** | **Total** | **p-value*** |
| **Obstructed breathing** | |  |  |  |
| HUU | 1168 (99.1) | 11 (0.9) | 1179 | 0.178 |
| HEU | 193 (100) | 0 (0) | 193 |  |
| **Respiratory distress** | |  |  |  |
| HUU | 1057 (89.7) | 122 (10.3) | 1179 | 0.182 |
| HEU | 179 (92.8) | 14 (7.2) | 193 |  |
| **Cyanosis** |  |  |  |  |
| HUU | 1169 (99.2) | 10 (0.8) | 1179 | 0.634 |
| HEU | 192 (99.5) | 1 (0.5) | 193 |  |
| **Convulsions** |  |  |  |  |
| HUU | 1146 (97.2) | 33 (2.8) | 1179 | 0.526 |
| HEU | 186 (96.4) | 7 (3.6) | 193 |  |
| **Severe anemia** |  |  |  |  |
| HUU | 1159 (98.3) | 20 (1.7) | 1179 | 0.887 |
| HEU | 190 (98.5) | 3 (1.5) | 193 |  |
| **Severe dehydration** | |  |  |  |
| HUU | 1150 (97.5) | 29 (2.5) | 1179 | 0.745 |
| HEU | 189 (97.9) | 4 (2.1) | 193 |  |
| **Shock** |  |  |  |  |
| HUU | 1172 (99.4) | 7 (0.6) | 1179 | 0.146 |
| HEU | 190 (98.5) | 3 (1.5) | 193 |  |
| **Profuse watery diarrhea** | |  |  |  |
| HUU | 1111 (94.2) | 68 (5.8) | 1179 | 0.970 |
| HEU | 182 (94.3) | 11 (5.7) | 193 |  |
| **Impaired consciousness** | |  |  |  |
| HUU | 1154 (97.9) | 25 (2.1) | 1179 | 0.679 |
| HEU | 188 (97.4) | 5 (2.6) | 193 |  |
| **Vomits everything** |  |  |  |  |
| HUU | 1156 (98.1) | 23 (1.9) | 1179 | 0.560 |
| HEU | 188 (97.4) | 5 (2.6) | 193 |  |

*****Used chi-square and Fisher’s exact test

# **Supplemental Figures**

**
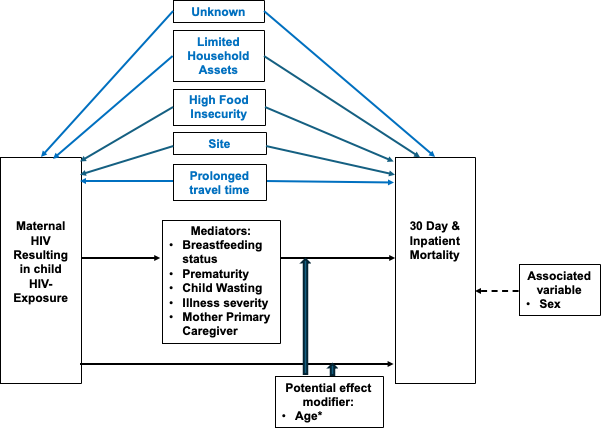
**

**Supplemental Figure 1. Directed acyclic graph illustrating hypothesized relationships between various clinical and sociodemographic variables, HIV-exposure, and 30 day and inpatient mortality.** Blue arrows indicate potential confounding variables; black arrows indicate potential mediators; dotted arrow indicates variables associated with the outcome; block arrows indicate potential effect modifiers. *Age < 6 months was tested as an effect modifier and not found to have a significant interaction (p=0.9 and p = 0.87 for inpatient and 30-day mortality, respectively). Age was subsequently adjusted for based on categories of < 6 months, 6-12 months, and greater than 12 months. The interaction between wasting status and mortality was also not found to be significant (p = 0.46 and p=0.24 for inpatient and 30-day mortality respectively). An exploratory analysis was performed with wasting status included in the final model (see Results section “HIV exposure and mortality).


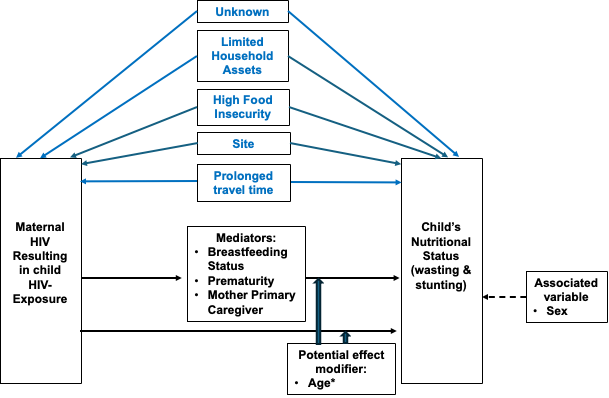


**Supplemental Figure 2. Directed acyclic graph illustrating hypothesized relationships between various clinical and sociodemographic variables, HIV-exposure, and child’s nutritional status.** Blue arrows indicate potential confounding variables; black arrows indicate potential mediators; block arrows indicate potential effect modifiers. The dotted arrow indicates variables associated with the outcome. Age < 6 months was tested as an effect modifier and was not found to have a significant interaction (p=0.8 and p=0.4 for wasting and stunting, respectively). *Age was subsequently adjusted for based on categories of < 6 months, 6-12 months, and greater than 12 months.

**

**Supplemental figure 3: Histogram of duration of hospitalization by HIV exposure category**

Length of hospital stay analysis was restricted to survivors and also excluded those who left against medical advice or absconded. The median length of hospital stay among all study participants was 5 days.


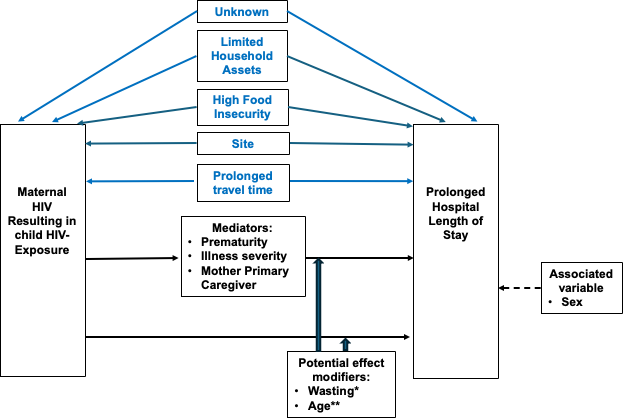


**Supplemental Figure 4. Directed acyclic graph illustrating hypothesized relationships between various clinical and sociodemographic variables, HIV-exposure, and hospital length of stay.** Blue arrows indicate potential confounding variables; black arrows indicate potential mediators; block arrows indicate potential effect modifiers. The dotted arrow indicates variables associated with the outcome. *Wasting status was tested as an effect modifier and not found to have a significant interaction (p=0.1). **Age < 6 months was tested as an effect modifier and not found to have a significant interaction (p= 0.13). Age was subsequently adjusted for based on categories of < 6 months, 6-12 months, and greater than 12 months.


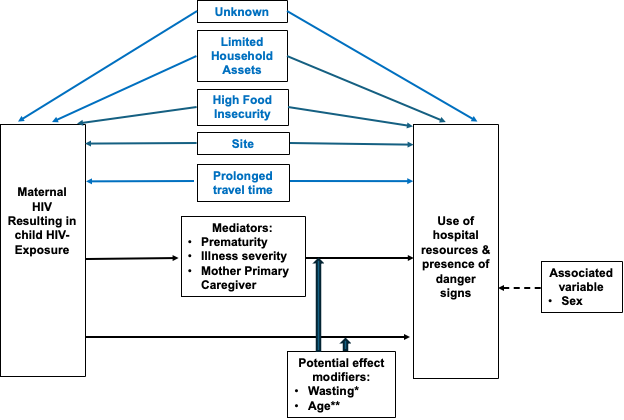


**Supplemental Figure 5. Directed acyclic graph illustrating hypothesized relationships between various clinical and sociodemographic variables, HIV-exposure, and use of hospital resources, and presence of danger signs.** Blue arrows indicate potential confounding variables; black arrows indicate potential mediators; block arrows indicate potential effect modifiers. The dotted arrow indicates variables associated with the outcome. *Wasting status was tested as an effect modifier and not found to have a significant interaction with presence of danger signs (p=0.86). **Age < 6 months was tested as an effect modifier and not found to have a significant interaction with presence of danger signs (p=0.55). Age was subsequently adjusted for based on categories of < 6 months, 6-12 months, and greater than 12 months.

**
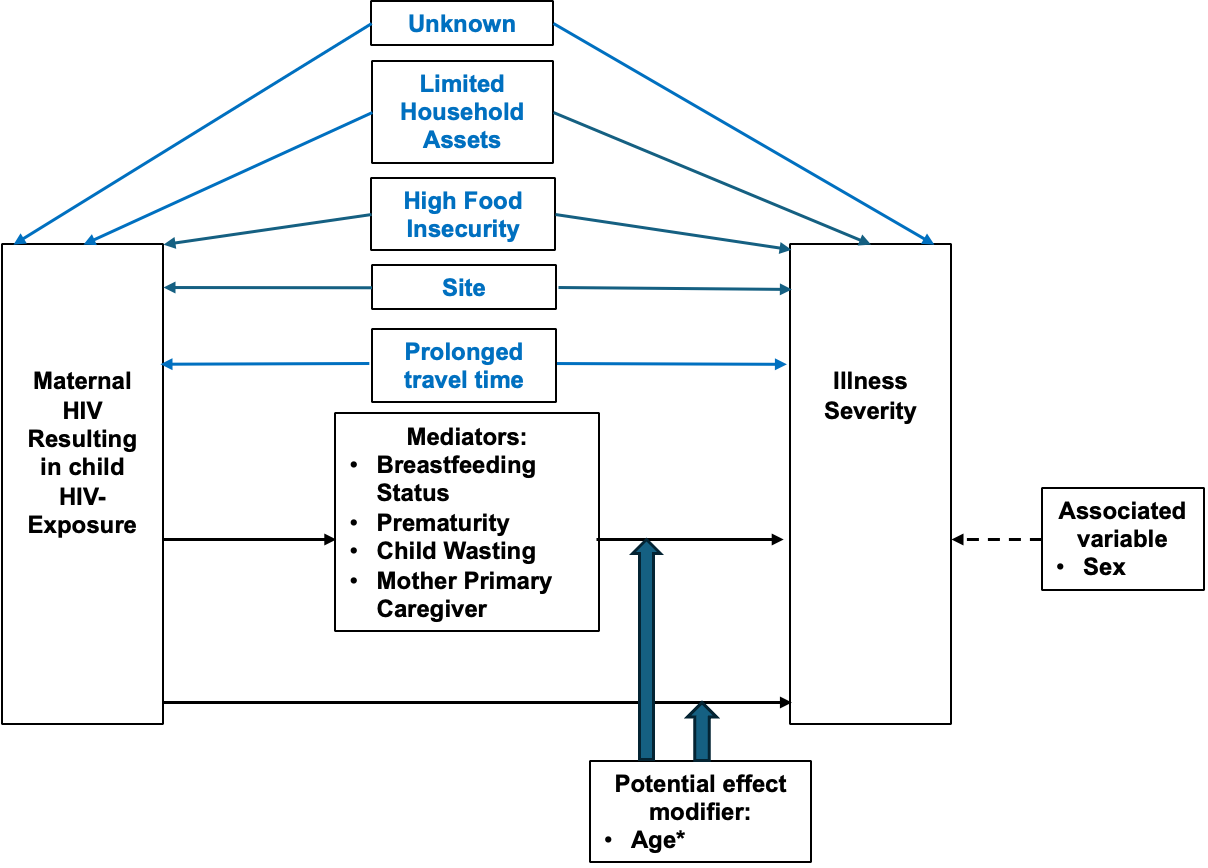
**

**Supplemental Figure 6. Directed acyclic graph illustrating hypothesized relationships between various clinical and sociodemographic variables, HIV-exposure, and illness severity at enrollment.** Blue arrows indicate potential confounding variables; black arrows indicate potential mediators; and block arrow indicates potential effect modifiers; block arrows indicate potential effect modifiers. The dotted arrow indicates a variable associated with the outcome. *Age < 6 months was tested as an effect modifier and found to have a significant interaction (p=0.013); a stratified analysis was subsequently performed.

**
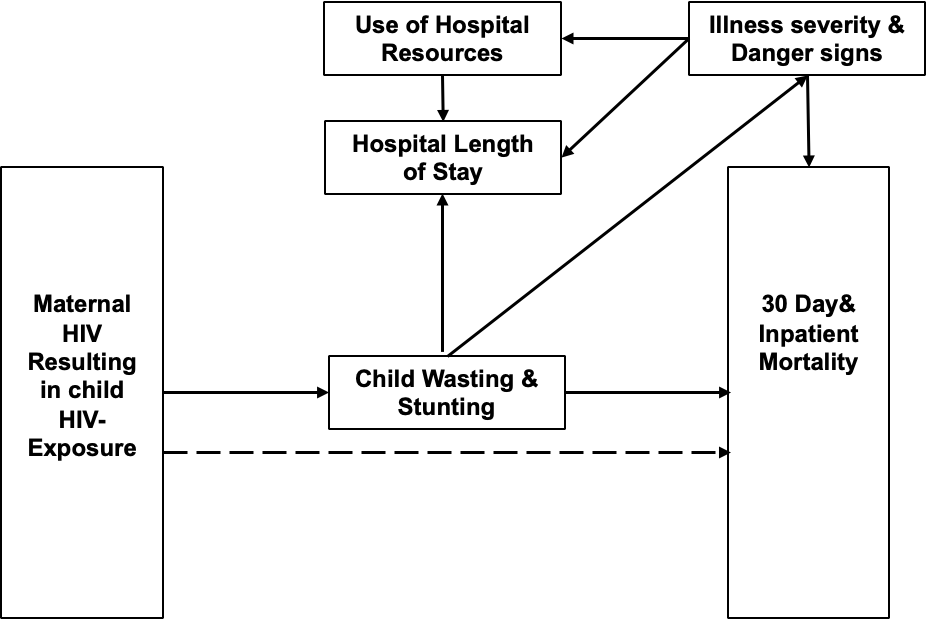
**

**Supplemental Figure 7. Directed acyclic graph illustrating hypothesized relationships between HIV-exposure and study outcomes.** Solid lines represent relationships explored in this analysis. The dotted line represents untested variables that may influence the relationship between HIV-exposure and mortality.
